# Empowering Patients Through Digital Health Literacy and Access to Electronic Medical Records (EMRs) in the Developing World

**DOI:** 10.7759/cureus.57527

**Published:** 2024-04-03

**Authors:** Sunder Sham, Sheena Shiwlani, Sanjay Kirshan Kumar, Prinka Bai, Ahmed Bendari

**Affiliations:** 1 Pathology and Laboratory Medicine, Lenox Hill Hospital, New York, USA; 2 Pathology, Mount Sinai Hospital, New York, USA; 3 Gastroenterolgy, Sindh Institute of Urology and Transplantation, Karachi, PAK; 4 School of Nursing, University at Buffalo, Buffalo, USA; 5 Pathology, Northwell Health/Lenox Hill Hospital, New York, USA

**Keywords:** healthcare transition, developing nation, digital health literacy, emr adoption barriers, emr

## Abstract

This editorial discusses the transformative potential of digital health literacy and the critical role of electronic medical records (EMRs) in promoting patient empowerment in the healthcare landscape of developing countries. It examines the impact of digital media in healthcare, noting its ability to both democratize access to information and services and pose risks of misinformation among populations with limited health literacy. The discussion includes an overview of key literacy components critical for effectively navigating the digital healthcare ecosystem. Our article highlights the critical role of EMR in facilitating a patient-centered care (PCC) model, with a special emphasis on making EMR systems accessible and user-friendly for vulnerable groups in developing countries. The core aim of our study is twofold: First, it sheds light on the significant challenges - be they technical, financial, or infrastructural - that obstruct the adoption of sophisticated EMR systems in these areas. Second, it explores the essential aspect of digital health literacy, advocating for its improvement as a vital step toward enabling patients to effectively engage with their medical records. By addressing these key issues, our study seeks to illustrate how enhancing digital health literacy, alongside increasing the accessibility of EMR systems, can empower patients in the developing world to actively participate in their healthcare processes. This dual focus aims to contribute to the broader discourse on improving healthcare outcomes through more inclusive and patient-centered approaches, particularly in settings that are currently underserved by modern healthcare technologies. In conclusion, the editorial advocates for a concerted effort toward creating a more inclusive and empowered healthcare paradigm. It suggests integrating PCC principles, tailoring EMR systems to diverse needs, and enhancing digital health literacy as strategies to harness digital health innovations for better healthcare outcomes and equity. It emphasizes the importance of ongoing investment in education, technology, and policy to fully leverage digital health solutions in the developing world.

## Editorial

For the past few years, digital media has become an important part of almost every stratum of daily life, where people are found to give a major share of their day to its use. The popularity of digital media has risen to incredible heights, and so has its involvement in the healthcare industry. It has made significant improvements in making advanced healthcare services and knowledge more accessible than ever before. Unfortunately, digital health or e-health can play a draconic role for people with limited health literacy by spreading factually incorrect information. In fact, limited health literacy is found to be closely associated with poorer health outcomes, such as increased emergency care use and hospitalization risks, making it an even more problematic issue than it was previously acknowledged. Therefore, to empower patients, especially in third-world countries where health education is not accessible, with the latest digital health technologies, one fundamental skill should be continuously developed: digital health literacy.

**Figure 1:**
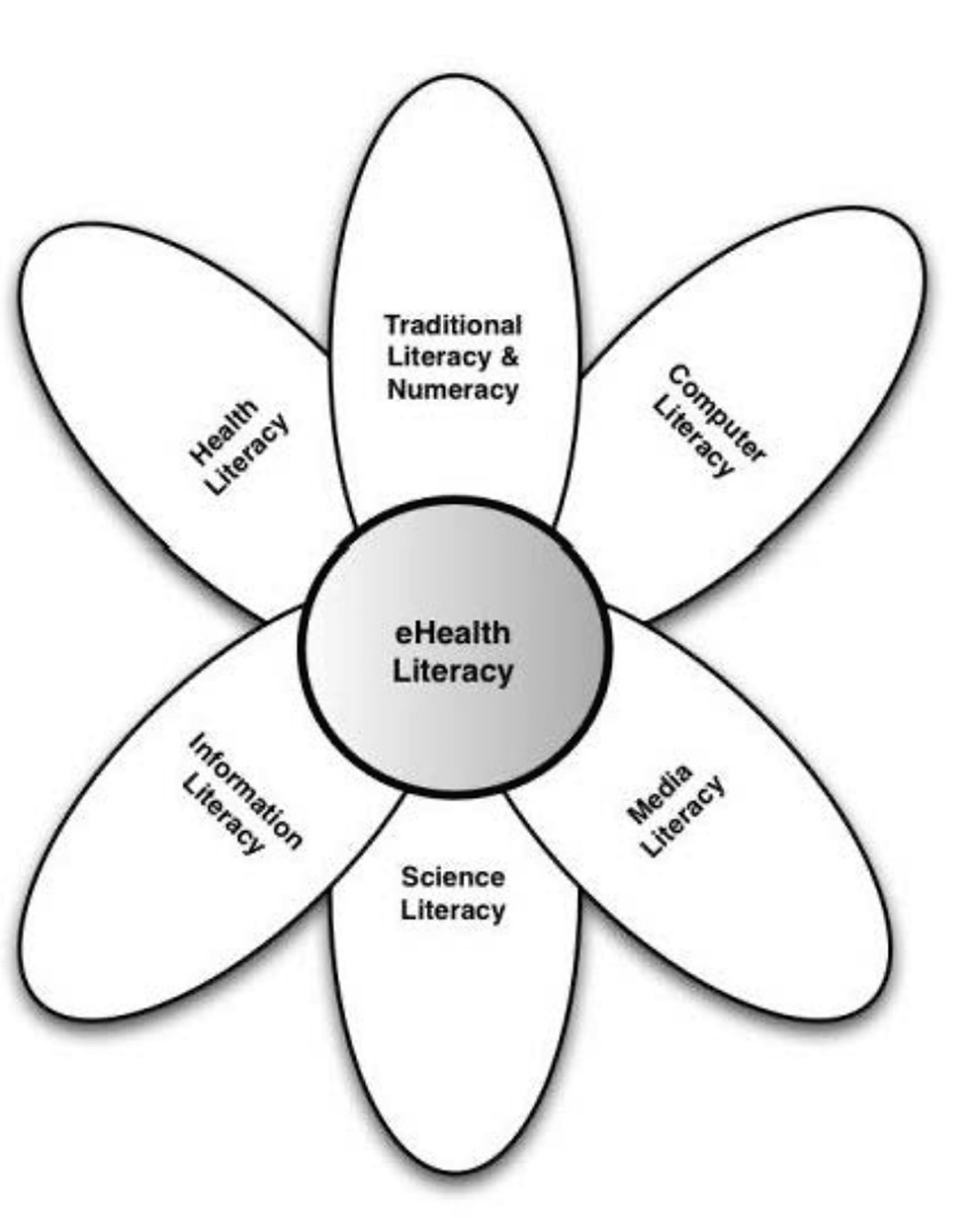
The conceptual lily model of digital health literacy Norman and Skinner's model, depicting the six components essential for digital health literacy: traditional literacy, media literacy, science literacy, information literacy, computer literacy, and health literacy [1].

Digital health literacy refers to people's ability to find relevant health information among the various digital databases distributed across the web, understand that particular piece of information, and finally use it to improve their overall health. Norman and Skinner beautifully described digital health literacy in their conceptual lily model (Figure 1), which divides the whole concept into six small parameters: traditional literacy and numeracy, media literacy, science literacy, information literacy, computer literacy, and finally health literacy [1]. Each of these components has its own importance, and a basic understanding of each is important for developing a particular skill set to access digital health services in a much better way.

Fortunately, with the rising popularity of digital health tools, achieving the benchmark of better health outcomes and overall well-being has never been easier, and the time has never been more opportune to revolutionize the whole healthcare framework, making it more focused on patient care, an approach that has been addressed as patient-centered care (PCC) in the developing world. Under the concept of PCC, patients are considered equal partners in healthcare, ultimately building a cooperative relationship between patients and physicians [2]. This has demonstrated that patients are not limited to their passive role as disease-carrying entities. Rather, they should actively contribute to decision-making about their health to achieve safe, high-quality, and cost-effective healthcare not offered previously in the absence of digital health literacy. One of the most important components that strengthen both DHL and PCC concepts in digital healthcare is electronic medical records or EMRs.

The current state of EMRs in developing countries

An EMR system is a real-time patient digital health record system that facilitates patient experience by providing a better user interface, showing every investigation and information using special tabs that are easily understandable by the audience. Recent studies indicated that EMRs are directly linked to the expectation, perception, and satisfactory understanding of their medical records and the delivery of healthcare services [3]. However, in many developing nations, ensuring equal access to these digital health records remains a challenge, especially for vulnerable groups like women and the elderly. These populations often encounter obstacles in accessing both digital health resources and traditional paper-based medical records due to a combination of limited digital literacy, economic barriers, and infrastructure deficits.

Highlighting these challenges underscores the importance of devising strategies that can overcome these barriers, ensuring that all individuals have the means to manage their health effectively. The ubiquity of smartphones and the increasing proficiency in using social media among older adults and women suggest a track toward making EMR apps more accessible and user-friendly. Furthermore, the widespread usage of smartphones in developing countries emphasizes the feasibility of integrating EMR apps into everyday healthcare practices. This not only enhances patient engagement but also lays the groundwork for a more inclusive and patient-centered healthcare system. Therefore, advocating for improved access to EMR, coupled with efforts to enhance digital health literacy, represents a transformative approach toward empowering patients, both in the developing world and beyond.

EMRs have fundamentally changed healthcare management in developed countries like the United States of America, offering numerous benefits for data handling and patient care. However, the adaptation and effective use of EMRs are not without challenges. Privacy and security concerns top the list, with the need for stringent measures to protect sensitive patient information from breaches and cyberattacks. In addition, the lack of interoperability between various EMR systems poses a significant barrier, especially for smaller healthcare facilities and those in recourse-limited settings. Resistance from healthcare providers often due to the steep learning curve and the time investment required to master these systems, along with the critical importance of data quality and accuracy, further complicates matters. Technical issues can lead to system downtimes, disrupting clinical workflows and patient care. Moreover, ensuring compliance with legal and regulatory standards presents an ongoing challenge.

EMRs and their challenges in developing countries

Indeed, the intricacies of introducing advanced EMR systems into less developed contexts merit thorough exploration. These systems, while prevalent in American healthcare settings, may encounter unique hurdles when applied in regions with different infrastructural, socioeconomic, and technological landscapes. For example, many regions in the developing world lack the necessary infrastructure to support advanced EMR systems. Issues, such as unreliable electricity supply, limited Internet connectivity, and insufficient hardware, may hinder the effective deployment and utilization of such technologies. In addition, the initial investment in software licenses, hardware, training, and ongoing technical support may surpass the budgets of many institutions. Therefore, while developing policies to support the technical and financial aspects of EMR system deployment, it is equally crucial to include measures that ensure patients have the digital health literacy necessary to access, understand, and benefit from their medical records.

However, when considering the risks associated with EMRs, the alternative should not also be overlooked - restricting patient access to their health care. Most of the information mentioned above clearly demonstrates that when patients are discouraged from understanding their health notes, it negatively impacts their overall health status [4]. In addition, it has also been noted that making patients unaware of their present health condition makes them more anxious and irritable, ultimately damaging the whole ideology of PCC in digital healthcare. Therefore, instead of making patient records inaccessible, the better solution will be to implement strategies like improving digital health literacy, which helps patients better understand their health records and minimize the risks and challenges that come with the implementation of e-health.

Interventions to effective EMR implementation in the developing world

EMRs hold immense potential for improving healthcare delivery in developing countries. However, their successful implementation necessitates a strategic approach tailored to the unique challenges faced in such contexts. For example, to address interoperability challenges, these countries should prioritize solutions that promote seamless integration of healthcare activities and departments. This involves providing comprehensive training programs and ongoing support to facilitate the transition from paper-based to electronic systems [5]. Another critical aspect that needs to be addressed during the successful implementation of EMRs is technological limitations. Therefore, only electronic records or software compatible with existing technology infrastructure should be selected. Later on, with the passing years, increased communication between healthcare providers and IT project managers will help healthcare authorities overcome organizational barriers, and software with even the latest and more advanced features will be successfully implemented [5]. Developing countries must also prioritize confidentiality, integrity, access control, and audit trail mechanisms to safeguard electronic records effectively, for which they can seek guidance and support from experienced nations to design foolproof data preservation protocols. Ultimately, the involvement of all stakeholders holds the key, as the participation of healthcare professionals, administrators, and patients in decision-making processes fosters stakeholder buy-in and acceptance of EMR systems.

Conclusion

The transformative potential of digital health literacy and access to EMRs in the developing world cannot be overstated. As digital media becomes increasingly intertwined with healthcare, it offers unparalleled opportunities for patient empowerment and improved health outcomes. The integration of PCC principles and the localization of EMR systems offer promising avenues for enhancing accessibility and usability, particularly in developing regions with diverse linguistic backgrounds. While concerns about patient anxiety and information comprehension persist, restricting access to healthcare records is not a viable solution. Instead, efforts should focus on enhancing digital health literacy and fostering a collaborative approach between patients and healthcare providers. Moving forward, continued investment in education, technology, and policy frameworks will be essential to harness the full potential of digital health solutions and ensure equitable access to quality healthcare for all. In conclusion, digital healthcare has incredible potential to revolutionize the basics of patient care. However, to exploit their full potential, patients need to be empowered with digital health literacy and tools like EMRs. Through these, both the quality of patient care and patient experience can be improved toward better health outcomes.
